# Total polyp number may be more important than size and histology of polyps for prediction of metachronous high-risk colorectal neoplasms

**DOI:** 10.1186/s12876-022-02177-1

**Published:** 2022-03-02

**Authors:** Hyuk Yoon, Cheol Min Shin, Young Soo Park, Nayoung Kim, Dong Ho Lee

**Affiliations:** 1 Department of Internal Medicine, Seoul National University Bundang Hospital, 300 Gumi-dong, Bundang-gu, Seongnam, Gyeonggi-do 463-707 South Korea; 2 Department of Internal Medicine and Liver Research Institute, Seoul National University College of Medicine, Seoul, South Korea

**Keywords:** Colorectal neoplasm, Risk factors, Metachronous

## Abstract

**Background:**

To evaluate whether the risk of metachronous high-risk colorectal neoplasm (HR-CRN) differs according to the indication for surveillance colonoscopy.

**Methods:**

Patients who underwent polypectomy or endoscopic resection of colorectal neoplasms were enrolled and classified into three groups according to the indication for surveillance colonoscopy: advanced colorectal neoplasm (ACRN: adenoma ≥ 10 mm, adenoma with high-degree dysplasia and/or villous component), advanced serrated polyps (ASP: hyperplastic polyp or sessile serrated lesion ≥ 10 mm, traditional serrated polyp), and high-risk polyps (HRP: 3 or more adenomas or serrated polyps). The primary outcome was the development of metachronous HR-CRN, defined as ACRN, ASP, or HRP at the first follow-up colonoscopy.

**Results:**

In total, 367 patients were enrolled (ACRN group: N = 264; ASP group: N = 33; HRP group: N = 70). Among the 160 patients who underwent follow-up colonoscopy, 28 (18%) had HR-CRN. In univariable analysis, indication for surveillance colonoscopy was not found to be associated with the development of metachronous HR-CRN. Instead, the total polyp number at index colonoscopy showed a positive association with the risk of metachronous HR-CRN in trend analysis (*p* = 0.001). In multivariable analysis, the presence of 5 or more polyps at index colonoscopy was found to be associated with the risk of metachronous HR-CRN (OR, 2.575, *p* = 0.049) after adjusting for risk factors, such as obesity, diabetes, and smoking.

**Conclusions:**

The risk of metachronous HR-CRN did not differ according to the main indications for surveillance colonoscopy. The presence of 5 or more polyps at index colonoscopy was the only risk factor for metachronous HR-CRN.

## Background

Strategies for surveillance colonoscopy in patients who undergo colonoscopic removal of neoplastic polyps are important clinical issues. Although the suggested follow-up interval for surveillance colonoscopy varies with guidelines, a shorter follow-up interval is generally recommended in cases of high-risk colorectal neoplasms [1–3]. Two risk categories for colorectal neoplasms are commonly described in the published literature—one is based on the size and histology of the neoplasm, while the other is based on the number of neoplasms [2]. Advanced adenomas, usually defined as adenomas ≥ 10 mm in size and those with high-degree dysplasia and/or a villous component, are representative of high-risk colorectal neoplasm. The presence of more than two adenomas is another risk factor for metachronous colorectal neoplasms. As the clinical importance of serrated lesions has been highlighted, serrated polyps ≥ 10 mm in size or with dysplasia are also considered high-risk colorectal neoplasms [4].

In South Korea, surveillance colonoscopy at 3 years after colorectal polypectomy is recommended for all patients who have had these types of polyps [5]. However, it is not clear whether the risk of metachronous colorectal neoplasm is the same in these heterogeneous groups. Considering this problem, the recent guidelines by the U.S. Multi-Society Task Force propose detailed follow-up intervals stratified by each risk factor [2]. For example, they state that in addition to the number of adenomatous polyps, the number of sessile serrated lesions must also be counted when evaluating the risk of metachronous colorectal polyps. However, in everyday practice, because there are too many factors to be considered, many clinicians tend to count the total number of neoplastic polyps without differentiating between adenomatous and sessile polyps when deciding the follow-up interval of surveillance colonoscopy in patients who undergo colorectal polypectomy.

In this study, we aimed to evaluate whether the risk of metachronous colorectal neoplasm is different among the main risk factors categorized by size, histology, and the total number of removed polyps. In particular, we wanted to evaluate whether there is a specific group that needs surveillance colonoscopy at 1 year after index colonoscopy.

## Methods

### Patients

All work was carried out in compliance with the Ethical Principles for Medical Research Involving Human Subjects outlined in the Helsinki Declaration in 1975 (revised in 2000). All subjects provided informed consent, and this study was approved by the institutional review board of the Seoul National University Bundang Hospital (B-1204/152-004). Between August 2012 and September 2017, individuals in whom polyps were found at the screening colonoscopy, and who underwent snare polypectomy or endoscopic resection of colorectal neoplasms at the Seoul National University Bundang Hospital were enrolled. They were classified into three groups according to the characteristics of the removed polyps: the advanced colorectal neoplasm (ACRN), advanced serrated polyps (ASP), and high-risk polyps (HRP) groups. ACRN was defined as adenomas ≥ 10 mm in size, adenomas with high-degree dysplasia and/or villous component, and early cancer. An ASP was defined as a hyperplastic polyp or sessile serrated lesion ≥ 10 mm in size or with dysplasia, or a traditional serrated polyp of any size. HRPs were defined as three or more adenomas or serrated polyps that did not fall into the two categories mentioned earlier. Patients who had both ACRN and ASP were classified into the ACRN group. Patients were also classified into four groups according to the total number of colon polyps: 1–2, 3–4, 5–9, and 10 or more, referring to previous studies [6, 7]. Non-neoplastic polyps, such as inflammatory polyps, were not considered in this study. The exclusion criteria for patients were as follows: (1) age > 75 years; (2) a diagnosis of familial adenomatous polyposis, hyperplastic polyposis, or inflammatory bowel disease; (3) personal history of colorectal cancer; (4) inadequate bowel preparation (Boston Bowel Preparation Scale score < 6) [8] or failed cecal intubation; (5) presence of only one or two adenomas or serrated polyps that did not correspond to both ACRN and ASP; and (6) additional surgery after polypectomy for submucosal colorectal cancer. We excluded patients over 75 years of age because it was not clear whether the benefit of surveillance colonoscopy outweighs procedure-related risks in this age group [2].

On the day of polypectomy or endoscopic resection of the colorectal neoplasms, data regarding known risk factors for colorectal neoplasms, such as family history, obesity, comorbidities, medications, and lifestyle, were collected for each patient using a survey [9, 10]. Obesity was defined as a body mass index ≥ 25 kg/m^2^. Visceral obesity was defined as waist circumference ≥ 90 cm in men and ≥ 85 cm in women based on the Korean Society for the Study of Obesity guidelines [11].

Our endoscopy unit has been accredited by Korean Society of Gastrointestinal Endoscopy to perform high-quality endoscopy practices [12]. All colonoscopic procedures were performed following a standard protocol such as adequate bowel preparation, cecal intubation, and enough withdrawal time. All polypectomies and endoscopic resections were performed by highly experienced endoscopists specializing in gastrointestinal diseases. All endoscopists had more than 5000 colonoscopy experiences. All procedures were performed with a standard single-channel endoscope (CF-Q260AL, Olympus Optical Co, Ltd, Tokyo, Japan). After polyp removal, the final pathologic report was carefully reviewed. If the inclusion criteria were present, surveillance colonoscopy 1 year later was recommended. The primary outcome was the development of metachronous high-risk colorectal neoplasm (HR-CRN), defined as any ACRN, ASP, or HRP at the first follow-up colonoscopy. Multivariable logistic regression was performed to evaluate the risk factors for metachronous HR-CRN.

### Statistical analysis

The baseline demographics of the patients in the three groups were compared using Fisher’s exact test. For variables that differed significantly among the three groups, post-hoc pairwise comparisons were performed, and a *p* value of 0.017 was considered to indicate statistical significance (accounting for a Bonferroni correction). For all other analyses, statistical significance was set at a 2-sided *p* value of < 0.05. Trend analysis was performed using a Wilcoxon-type test for trend. Possible clinical predictors of metachronous HR-CRN were analyzed using univariable logistic regression, and variables with a *p* value < 0.2 were then included in the multivariable logistic regression analysis. All analyses were performed using STATA version 16.0 (StataCorp LLC, Texas, USA).

## Results

### Baseline characteristics of the patients

Among a total of 508 patients, 141 patients were excluded after the final pathologic reports were checked. The most common reason for exclusion was that the size of the polyps was smaller than initially expected. Finally, 367 patients were included in the study. The proportion of patients in the ACRN, ASP, and HRP groups was 72%, 9%, and 19%, respectively. Table 1 shows the baseline demographics of the patients in the three groups. The age distributions of the three groups were different. In post-hoc analysis, the proportion of patients < 50 years old was higher in the ASP group than in the other two groups (*p* = 0.002 vs. ACRN group, *p* = 0.019 vs. HRP group). The proportion of patients with comorbidities also differed among the three groups. The proportion of patients with hypertension was lower in the ASP group than in the other two groups (*p* = 0.039 vs. ACRN group, *p* = 0.006 vs. HRP group). The proportion of patients with diabetes was also lower in the ASP group than in the other two groups (*p* = 0.021 vs. ACRN group, *p* = 0.002 vs. HRP group). The proportion of patients taking aspirin was lower in the ASP group than in the HRP group (*p* = 0.015). The proportion of patients who were taking alcohol was higher in the HRP group than in the other two groups (*p* < 0.001 vs. ACRN group, *p* = 0.006 vs. ASP group). When we divided all patients into younger patients (aged < 50 years) and older patients (aged ≥ 50 years) regardless of the initial three groups, younger patients had less hypertension and diabetes than older patients (*p* < 0.001 and *p* = 0.004, respectively). The proportion of patients taking aspirin was also lower in younger patients than in older patients (*p* = 0.001).

**Table 1:** Baseline demographics of the included patients (N = 367)

	ACRN group (N = 264)	ASP group (N = 33)	HRP group (N = 70)	*p* value
Age < 50 years	51 (19%)	15 (45%)	15 (21%)	0.006
Male	180 (68%)	22 (67%)	55 (79%)	0.207
Obesity	101 (39%)	10 (32%)	32 (46%)	0.353
Visceral obesity	36 (16%)	3 (10%)	13 (21%)	0.449
Comorbidity				
Hypertension	77 (29%)	4 (12%)	28 (40%)	0.012
Diabetes	37 (14%)	0	16 (23%)	0.004
Dyslipidemia	50 (19%)	4 (12%)	15 (21%)	0.531
Medications				
Aspirin	25 (9%)	0	11 (16%)	0.030
Statins	34 (13%)	3 (9%)	13 (19%)	0.361
Metformin	21 (8%)	0	8 (11%)	0.116
Current smoker	82 (31%)	8 (25%)	19 (27%)	0.700
Alcohol intake	137 (52%)	16 (49%)	54 (77%)	< 0.001
Family history of CRC	27 (10%)	4 (13%)	7 (10%)	0.883

*ACRN* advanced colorectal neoplasm, *ASP* advanced serrated polyps, *HRP* high-risk polyps, *CRC* colorectal cancer

Table 2 shows the characteristics of the colorectal polyps at index colonoscopy. The most common inclusion criterion was adenoma ≥ 10 mm in size (66.9%), followed by HRP and adenoma with a villous component. Among ASP, there were lesions with dysplasia.

**Table 2:** Characteristics of colorectal polyps at index colonoscopy

Group	Enrollment criterion	N (%)
ACRN		
	Adenoma ≥ 10 mm	253 (66.9)
	Adenoma with villous component	78 (20.6)
	Adenoma with high-degree dysplasia	45 (11.9)
	Intramucosal or submucosal cancer	45 (11.9)
ASP		
	Hyperplastic polyp ≥ 10 mm	5 (1.3)
	Sessile serrated lesion ≥ 10 mm	30 (7.9)
	Traditional serrated polyp	19 (5.0)
Total number of polyps		
	1–2	171 (45.2)
	3–4	109 (28.8)
	5–10	86 (22.8)
	> 10	12 (3.2)

*ACRN* advanced colorectal neoplasm, *ASP* advanced serrated polyps

### Follow-up colonoscopy after polyp removal

**Fig. 1:**
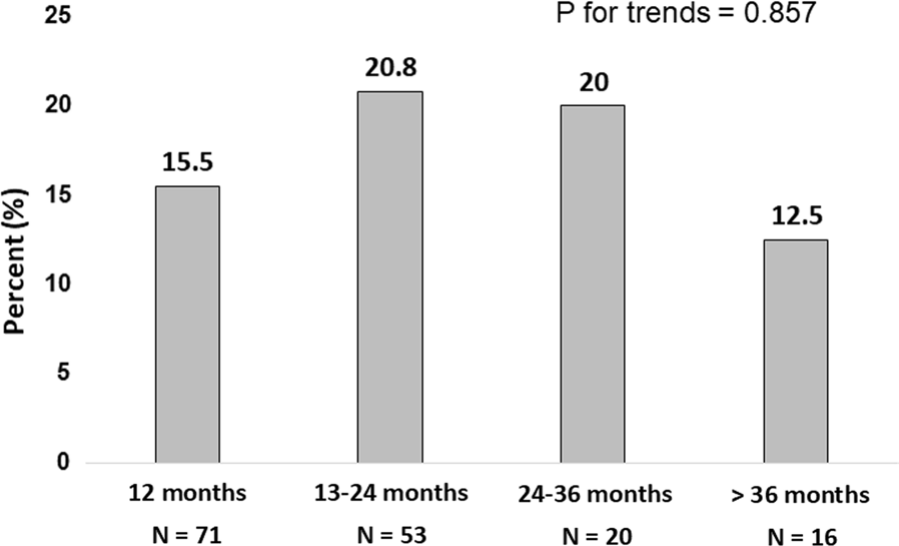
Incidence of metachronous high-risk colorectal neoplasms according to the time point of surveillance colonoscopy

Of the 367 patients included in the study, 220 underwent follow-up colonoscopy. We excluded 60 patients who underwent follow-up colonoscopy less than 12 months after index colonoscopy because it is possible that the polyps found on follow-up colonoscopy were polyps missed on index colonoscopy. The reasons that the patients underwent follow-up colonoscopy less than 12 months were as follows: positive or uncheckable resection margin of the lesions at the final pathologic report (38%), request of patients who wanted to ensure no recurrence of intramucosal or submucosal cancer (28%), not apparent reason (20%), personal issues (14%). Finally, 160 patients were analyzed for the primary outcome. Metachronous HR-CRN was found in 28 patients (17.5%). ACRN, ASP, and HRP were found in 6 patients (3.8%), 4 patients (2.5%), and 20 patients (12.5%), respectively. Of the 20 patients who had multiple polyps, two also had ACRN. The recommended time interval from index colonoscopy to surveillance colonoscopy was 1 year; however, the actual time interval varied widely for some patients. Even though the proportion of patients who underwent surveillance colonoscopy at exactly 1 year was 32.3%, most patients (90%) underwent follow-up colonoscopy within 3 years after the index colonoscopy. No trend in the proportion of patients with metachronous HR-CRN was observed based on the time interval of surveillance colonoscopy (*p* value for trends = 0.857) (Fig. 1).

### Risk factors for metachronous high-risk colorectal neoplasms

**Fig. 2:**
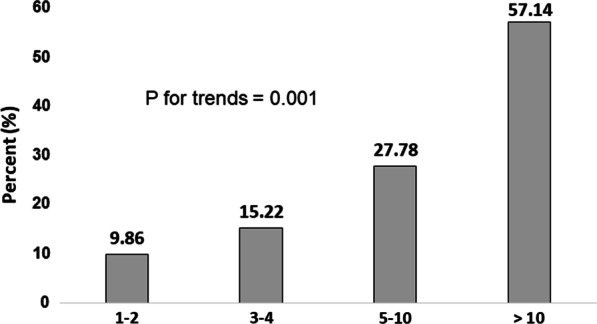
Incidence of metachronous high-risk colorectal neoplasms according to the total number of polyps at index colonoscopy

In univariable logistic regression analysis, the odds ratios (ORs) for metachronous HR-CRN were not significantly different among the three groups (Table 3). Instead, regardless of the presence of ACRN or ASP at index colonoscopy, as the total number of polyps increased, the proportion of patients with metachronous HR-CRN also increased (Fig. 2); moreover, this trend was statistically significant (*p* = 0.001). Specifically, patients with 5 or more polyps at index colonoscopy had a significantly higher risk of metachronous HR-CRN (OR, 3.552; 95% CI, 1.522–8.290; *p* = 0.003). In multivariable logistic regression analysis, the presence of 5 or more polyps was an independent risk factor for metachronous HR-CRN after adjusting for other factors (OR, 2.575; 95% CI, 1.003–6.613; *p* = 0.049).

**Table 3:** Results of the logistic regression analysis for the prediction of metachronous high-risk colorectal neoplasms

Variables	Univariable			Multivariable		
	OR	95% CI	*p* value*	OR	95% CI	*p* value*
Age ≥ 50 years	1.543	0.494–4.823	0.456			
Female	1.2	0.483–2.980	0.694			
Obesity	0.817	0.355–1.882	0.636			
Visceral obesity	2.105	0.763–5.806	0.151	1.822	0.630–5.271	0.268
Hypertension	1.387	0.597–3.219	0.447			
Diabetes	2.255	0.833–6.102	0.109	1.571	0.519–4.758	0.424
Dyslipidemia	1.2	0.483–2.980	0.697			
Aspirin	0.431	0.095–1.960	0.276			
Current smoker	1.775	0.757–4.158	0.187	1.531	0.588–3.985	0.383
Alcohol intake	1.455	0.624–3.389	0.385			
Family history of CRC	1.240	0.325–4.729	0.757			
Inclusion criteria						
ACRN	1					
ASP	0.259	0.033–2.055	0.201			
HRP	0.827	0.285–2.405	0.728			
Polyps ≥ 5	3.552	1.522–8.290	**0.003**	2.575	1.003–6.613	**0.049**

*ACRN* advanced colorectal neoplasm, *ASP* advanced serrated polyps, *HRP* high-risk polyps, *CRC* colorectal cancer

*Boldface indicates statistical significance

## Discussion

There are two main types of neoplastic polyps that have the malignant potential to progress to colorectal cancer—conventional adenomatous polyps and serrated polyps. Adenomatous polyps can be further categorized according to the following features: size, degree of dysplasia, and proportion of villous components. Serrated polyps can be further categorized into hyperplastic polyps, sessile serrated lesions, and traditional serrated polyps [13]. Many guidelines use these intrinsic factors and the number of polyps to suggest the adequate follow-up interval for surveillance colonoscopy after colorectal polypectomy. However, in clinical practice, determining the proper follow-up interval for each patient is not so straightforward. For example, many patients have both adenomatous polyps and serrated polyps [14]. In case of a patient with both advanced adenomatous polyps and advanced serrated polyps, it is difficult to determine which polyp poses a higher risk of metachronous neoplasm and should therefore be prioritized for determining the follow-up interval of surveillance colonoscopy. In addition, it is also unclear whether the risk of metachronous colorectal neoplasm is as high in patients with multiple (≥ 3) but small (< 10 mm in size) adenomatous polyps without any high-risk histologic features as in patients with advanced adenomatous polyps. Therefore, we compared the risk of metachronous HR-CRN among the ACRN, ASP, and HRP groups and found that it was not significantly different among the three groups. Instead, the number of total polyps, regardless of size and other histologic features of each polyp, was independently associated with the risk of metachronous HR-CRN. The percentage of patients who had metachronous HR-CRN increased as the total number of polyps at index colonoscopy increased—metachronous HR-CRN was found in more than half (57%) of patients with more than 10 polyps. After adjusting for other factors, patients with multiple polyps (5 or more) had a higher risk of metachronous HR-CRN (OR 2.575). This result corresponds very well with that of a recent Spanish study, in which the presence of multiple polyps (3 or more adenomas and/or serrated polyps) was found to be a strong predictor of HR-CRN after index polypectomy [15]. The study also did not find any histological characteristics that increased the risk of metachronous HR-CRN. However, contrary to our study, bowel preparation was not evaluated.

A recent meta-analysis reported that there was no significant difference in the risk of metachronous ACRN between patients with serrated polyps and those with conventional adenomas [16]. However, because most studies included in this meta-analysis had not reported the results according to size or number of serrated polyps, this meta-analysis could not assess the comparative risk of metachronous ACRN between ACRN and ASP. In keeping with this, the U.S. Multi-Society Task Force on Colorectal Cancer recommends a 3-year surveillance interval not only for both ACRN and ASP, but also in cases with 5–10 adenomas and 5–10 serrated lesions. In the present study, ACRN and ASP were not found to be independent risk factors for metachronous HR-CRN when surveillance colonoscopy was performed mostly within 3 years of the index colonoscopy. Therefore, applying the current U.S. recommendations for ACRN and ASP to Korean subjects seems reasonable. It is not clear whether one of these two main categories of advanced neoplastic polyps should be prioritized over the other. Nevertheless, as the risk for metachronous HR-CRN was higher in patients with multiple polyps (5 or more) regardless of the size and histology of each polyp, more intensive surveillance (at intervals shorter than 3 years) deserves consideration for patients who have 5 or more polyps when counted by combining adenomas and serrated lesions. However, because no trend in the proportion of patients who had metachronous HR-CRN was observed depending on the time interval of surveillance colonoscopy in the 1–3-year range, it is still unclear whether surveillance colonoscopy at 1 year is beneficial in these high-risk patients. Evidence for 1 year follow-up for multiple adenomas (more than 10) in the current U.S. guideline stems from a Korean study [17]. However, in the present study, when we performed additional analysis, the presence of more than 10 polyps was not found to be a statistically significant independent risk factor for metachronous HR-CRN. Although, given that metachronous HR-CRN was found in 57% of patients with more than 10 neoplastic polyps, our study also suggests that shorter surveillance intervals are considerable for this patient group.

Interestingly, contrary to the other two groups, the proportion of patients below 50 years of age in the ASP group was as high as 45% in the present study. The proportions of patients with hypertension and diabetes and of patients taking aspirin were relatively low in the ASP group; this could be attributed to the relatively younger age distribution in the ASP group. Similar trends for age distribution of patients with serrated polyps were also found in a previous study performed at our institution [18], in which the prevalence of conventional adenomas at screening colonoscopy was found to increase sharply with age. In contrast, the prevalence of serrated polyps appeared to be relatively high among patients aged < 50 years. The reasons for these phenomena are unclear; however, considering the recent increase in colorectal cancer in young adults [19], these results suggest that we should pay more attention not to miss serrated polyps in younger individuals.

The present study has several strengths. This was a prospective study, and we analyzed the results after adjusting for important major risk factors for colorectal cancer, including obesity, comorbidities, family history, and drug use. Nevertheless, this study also had certain limitations. First, the quality of the endoscopic procedure may affect the incidence of metachronous HR-CRN. Even though highly experienced endoscopists performed all procedures in this study, we had not monitored the adenoma detection rate of these endoscopists. In addition, we did not use magnifying colonoscopy. Therefore, the appropriateness of polypectomy may not have been thoroughly evaluated. To avoid missed lesions being considered newly developed lesions, we excluded patients who underwent follow-up colonoscopy less than 12 months after index colonoscopy. However, because it is challenging to distinguish between missed polyps and newly developed polyps, it is still possible that some missed polyps were included in metachronous polyps. Second, information was not collected about whether endoscopic mucosal resection for large polyps (≥ 20 mm in size) was performed en-bloc or piecemeal and the morphological characteristics of these large lesions. However, we believe that most patients with incomplete polyp resection were excluded from this study because we excluded patients who underwent follow-up colonoscopy less than 12 months after index colonoscopy, and the main reason due to which they underwent follow-up colonoscopy so early was incomplete resection. Third, although the patients were prospectively enrolled in this study, some parts of the study design are retrospective. The cut-off value for 5 or more polyps as a risk factor for metachronous HR-CRN was not set before study initiation. Therefore, although some studies suggest 5 as a cut-off for the total number of polyps [2, 5, 6], this value cannot be an absolute cut-off to determine the risk of metachronous HR-CRN. Last, because information about previous colonoscopy procedures was not collected, one must be cautious when applying these results to the general population. We started this study to evaluate the risk factors for metachronous HR-CRN. Therefore, we collected various risk factors using a survey. However, we found later that information about the history of colonoscopy was missing from the questionnaire. Therefore, the history of colonoscopy in each patient might affect the results of this study.

## Conclusions

In conclusion, the presence of multiple neoplastic polyps (5 or more) was an independent risk factor for metachronous HR-CRN in patients who underwent colorectal polyp removal. Our results suggest that the total polyp number may be considered just as important, if not more important than the complex information about the size and histologic features of the polyps in determining the surveillance interval after colorectal polypectomy. To draw robust conclusions about surveillance colonoscopy at 1 year is beneficial in these high-risk patients, further prospective studies in a large cohort are needed.

## Data Availability

The datasets used and/or analysed during the current study are available from the corresponding author on reasonable request.

## Abbreviations

HR-CRN: High-risk colorectal neoplasm
ACRN: Advanced colorectal neoplasm
ASP: Advanced serrated polyps
HRP: High-risk polyps
